# Understanding the roles of the healthcare and child welfare systems in promoting the safety and well-being of children

**DOI:** 10.3389/fpsyt.2023.1195440

**Published:** 2023-05-30

**Authors:** Nicolette Joh-Carnella, Eliza Livingston, Miya Kagan-Cassidy, Ashley Vandermorris, Jennifer N. Smith, Daniel M. Lindberg, Barbara Fallon

**Affiliations:** ^1^Factor-Inwentash Faculty of Social Work, University of Toronto, Toronto, ON, Canada; ^2^The Hospital for Sick Children, Toronto, ON, Canada; ^3^Department of Paediatrics, University of Toronto, Toronto, ON, Canada; ^4^Departments of Emergency Medicine and Pediatrics, University of Colorado Anschutz Medical Campus, Aurora, CO, United States

**Keywords:** pediatrics, child abuse and neglect, reporting, child welfare, healthcare, Canada

## Abstract

**Introduction:**

The accurate identification and appropriate investigation of child maltreatment is a key priority for promoting the optimal health and development of children. Healthcare providers are often well-positioned professionals to report suspected child abuse and neglect, and, therefore, interact regularly with child welfare workers. Little research has examined the relationship between these two groups of professionals.

**Methods:**

We interviewed healthcare providers and child welfare workers in order to examine the referral and child welfare investigation processes to understand strengths and identify areas of improvement for future collaboration. Thirteen child welfare workers from child welfare agencies and eight healthcare providers from a pediatric tertiary care hospital in Ontario, Canada were interviewed to meet the study’s objectives.

**Results:**

Healthcare providers spoke about positive experiences making reports, factors impacting reporting decisions, areas for improvement (e.g., difficulties communicating, lack of collaboration, and disruption of therapeutic alliance), training, and professional roles. For interviews with child welfare workers, identified themes included healthcare professionals’ perceived expertise and understanding the role of child welfare. Both groups brought up the need for increased collaboration as well as systemic barriers and legacies of harm.

**Discussion:**

Our core finding was a reported lack of communication between the groups of professionals. Other identified barriers in collaboration included a lack of understanding of each other’s roles, hesitation for healthcare providers making reports, as well as legacies of harm and systemic inequities in both institutions. Future research should build on this examination by including the voices of healthcare providers and child welfare workers to identify sustainable solutions for increased collaboration.

## 1. Introduction

Child maltreatment is a significant public health concern associated with increased adverse physical health, mental health, and developmental outcomes, along with increased morbidity and mortality (1–3). Healthcare providers in Canada are mandated to report suspected child maltreatment and play an important role in the prevention, identification, and management of child maltreatment concerns (4–6). Data from the Ontario Incidence Study of Reported Child Abuse and Neglect 2018 (OIS-2018) indicate that child welfare investigations referred by healthcare professionals are more likely to be substantiated and involve more intrusive forms of child welfare involvement, compared to investigations referred by other sources (5, 7). In general, families who come into contact with the child welfare system are often struggling in multiple domains, including concerns with economic insecurity, precarious living conditions, intimate partner violence, as well as substance use and mental health issues for caregivers (7–9). Previous studies looking specifically at hospital-based referrals to child welfare have documented these household- and caregiver-related concerns (7, 10).

The child welfare and healthcare systems are in a unique and opportune position to work together to support families, provide resources aligned with their needs, and intervene in situations of suspected maltreatment. However, few studies have focused on how these systems interact to support families; extant literature tends to focus on healthcare providers’ experiences, with fewer studies examining the reception of reports made by healthcare providers within the child welfare system.

Studies looking at healthcare professionals’ experiences engaging child welfare have reported healthcare providers’ discomfort with and lack of confidence in reporting suspected child maltreatment (11–22). For example, one study examining healthcare professionals’ experiences seeking support from the child welfare system reported a lack of routine screening for maltreatment, discomfort with discussing maltreatment, and inadequate knowledge of community resources (1). A Canadian study of the experiences of resident physicians training in a variety of medical specialties (i.e., pediatrics, family medicine, emergency medicine, obstetrics and gynecology, and psychiatry) identifying and reporting child maltreatment found that residents felt they required further training on what constitutes child maltreatment and how to identify non-physical forms of maltreatment (16). Further, the authors highlighted that study participants reported confusion around the reporting process to child welfare services and what their role was following a report (16). It could be that physicians are well-positioned and yet ill-equipped to identify risk factors for child maltreatment in order to intervene early and hopefully mitigate the need for additional child welfare involvement.

Given the dearth of literature on this topic, the current paper fills an important knowledge gap with respect to interactions between the healthcare and child welfare systems. By interviewing both healthcare providers (including physicians and nurse practitioners) who make referrals to child welfare for suspected child maltreatment, as well as child welfare workers who receive these referrals, our objective was to holistically examine the referral and child welfare investigation processes to identify gaps for future intervention at both the provider and policy levels.

## 2. Materials and methods

### 2.1. Participants and sampling

We conducted two sets of interviews simultaneously to meet the study’s objectives: (1) interviews with child welfare workers with experience investigating cases referred to child welfare by healthcare professionals and (2) interviews with healthcare providers (i.e., staff physicians and nurse practitioners) who had made referrals to child welfare agencies for concerns of child maltreatment. Recruitment emails were sent out to eligible staff at two large child welfare agencies (government-funded agencies that receive and respond to reports of child maltreatment) in Ontario, Canada as well as at one tertiary care children’s hospital. The recruitment email instructed interested participants to contact the study team to arrange an interview. Following this initial identification of participants through purposive sampling, snowball sampling was used to recruit further participants. Specifically, following each interview, participants were asked by the research assistant conducting the interview to identify colleagues with relevant experience who might be interested in study participation. The potential participants were then sent individualized emails soliciting their interest in the study. In total, 13 child welfare workers and eight healthcare providers participated in the study.

### 2.2. Ethics approvals

Ethics approval for this study was obtained from the University of Toronto ethics board (protocol number: 41000) as well as from individual ethics boards of participating institutions (i.e., the tertiary care children’s hospital and child welfare agencies).

### 2.3. Data collection

Participants arranged interviews with research assistants via email, and interviews were conducted using Microsoft Teams video conferencing (Microsoft, Redmond, WA). One research assistant was responsible for interviewing all child welfare workers who participated in the study, while interviews with healthcare providers were conducted by a separate research assistant and the project lead. The purpose of the study and potential risks were identified to the participants prior to the interview. Consent was obtained from all participants before beginning the interview, including consent to participate in the study, conduction of the interview via Microsoft Teams, the use of the transcription function within Microsoft Teams, and a separate audio recording of the interview. All but one participant consented to the use of the Microsoft Teams transcription function and audio recording. The one participant that did not give consent did allow the research assistant to take handwritten notes. In one other interview, the audio recording malfunctioned. Data from those two interviews (both with child welfare workers) were used to support themes, but direct quotes from those interviews are not used in the current paper. All other interviews were transcribed verbatim using both the Microsoft Teams transcription function and the audio recording.

Interviews were approximately 30 min in length and were conducted using a semi-structured approach. The interview guide consisted of seven questions for child welfare workers and 12 questions for healthcare providers; questions were designed to be open-ended and had designated prompts to elicit further information from participants (see Appendix for the list of interview questions). Questions for healthcare providers focused on: training specific to assessing child maltreatment, experiences and implications of reporting suspected maltreatment to child welfare, awareness of resources outside of child welfare, and suggestions for how the healthcare and child welfare systems can collaborate to better support children and families. Questions for child welfare workers focused on: reasons why healthcare worker reports might lead to more intrusive child welfare interventions, experiences working with healthcare providers, and areas of improvement for collaborating with the healthcare system (23). Data collection continued until thematic saturation was reached, meaning participants began sharing similar information and data were not resulting in new themes (24).

### 2.4. Demographic characteristics

Following completion of their interviews, participants were emailed a request to complete a survey that included information on their current position, experience in their respective fields, and demographic information (i.e., age, gender, and race/ethnicity). Completion of this survey was voluntary. Demographic information was provided by 12 (of a total 13) child welfare workers and six (of a total eight) healthcare providers. See Table 1 for a detailed summary of the participant demographics.

**Table 1:** Demographic characteristics of study participants.

	*N*	%
Child welfare workers		
Current position		
Child welfare worker – investigations	6	50%
Child welfare worker – ongoing	3	25%
Child welfare worker – other	3	25%
Primary responsibility (current position)		
Clinical	5	42%
Leadership	0	0%
Other	6	50%
Number of years of practice		
0–5 years	0	0%
5–10 years	1	8%
>10 years	11	92%
Age		
21–30 years	1	8%
31–40 years	3	25%
41–50 years	2	17%
51–60 years	4	33%
60+ years	1	8%
Prefer not to say	1	8%
Gender		
Male	2	17%
Female	10	83%
Non-binary	0	0%
Prefer not to say	0	0%
Race/ethnicity*		
White	10	83%
Latin American	1	8%
Indigenous	1	8%
Prefer not to say	1	8%
*One worker selected two categories		
Healthcare providers		
Current position		
Physician	6	100%
Nurse practitioner	0	0%
Healthcare worker – other	0	0%
Primary responsibility (current position)		
Clinical	6	100%
Leadership	1	17%
Other	0	0%
Number of years of practice		
0–5 years	1	17%
5–10 years	3	50%
>10 years	2	33%
Age		
21–30 years	1	17%
31–40 years	4	67%
41–50 years	1	17%
51–60 years	0	0%
Prefer not to say	0	0%
Gender		
Male	0	0%
Female	6	100%
Non-binary	0	0%
Prefer not to say	0	0%
Race/ethnicity*		
White	4	57%
Japanese	1	14%
Chinese	1	14%
Prefer not to say	1	14%
* One worker selected two categories		

#### 2.4.1. Healthcare providers

All six healthcare providers who completed the demographic survey were physicians. Although two nurse practitioners participated in the study, they did not complete the demographic questionnaire. Each physician identified their role as being primarily clinical, and one physician indicated they also had a leadership role. One had been practicing for 1–5 years, three had been practicing for 5–10 years, and two had been practicing for over 10 years. Most respondents (four out of six) were in the 31–40 age range, all six respondents were female, and three identified as white.

#### 2.4.2. Child welfare workers

Half of the participating child welfare workers who responded to the demographic survey (six out of 12) primarily conducted investigations, representing the front-end of the child welfare service continuum. Eleven of the 12 survey respondents indicated they had over 10 years of experience. Most of the child welfare workers who participated in the study and provided their demographic information identified their gender as female (10 of 12 respondents) and their race as white (10 of 12 respondents).

### 2.5. Data analysis

We employed a constructivist thematic analysis approach for interview coding (25). A theoretical process was used, whereby the research team had an understanding of relevant literature when considering possible themes, and coding was conducted using NVivo software (25). One research assistant coded all interviews conducted with child welfare workers and a separate research assistant coded all interviews with healthcare providers. The project lead served as a secondary coder for nine interviews (four out of eight interviews with healthcare providers and five out of 13 interviews with child welfare workers). The researchers then met to discuss the identified codes, ensure they were consistent between coders, and collate these codes into relevant themes. All themes were reviewed by the study’s principal investigator, who holds a PhD in Social Work, and all themes from interviews with healthcare providers were additionally reviewed by two of the study’s co-investigators who are physicians. All study investigators have considerable experience in the healthcare and child welfare fields. This collaborative process ensured the trustworthiness of the analysis.

## 3. Results

Pertinent themes are described below.

### 3.1. Healthcare providers

#### 3.1.1. Theme 1: positive experiences

##### 3.1.1.1. Interactions with child welfare workers

Participating healthcare providers reported that, overall, their interactions with child welfare have been positive. Participants mentioned that good collaboration between healthcare providers and child welfare workers contributes to positive experiences, especially when caseworkers “feel like a part of the healthcare team” (HCW-8). In addition, participants noted child welfare involvement can positively impact children and families, particularly when workers are supportive and can connect caregivers to helpful resources such as those that address concrete needs (e.g., arranging transportation or providing cribs and car seats), as well as those aimed at meeting social needs like mental health supports and parenting classes. One participant summarized it as child welfare’s ability to “mobilize systems around the family” (HCW-2).

##### 3.1.1.2. Impact of reporting on healthcare providers’ therapeutic relationships with families

Some healthcare providers (3/8) mentioned that, in certain situations, making a report to child welfare had a positive impact on their relationship with a family. One participant shared an example of a family accessing needed support as a result of child welfare involvement, as the participant stated: “once this support was in place, actually their lives really changed for the better… I feel like they see me kind of as instrumental in that improvement actually, because I made the call” (HCW-8). Participants shared they almost always informed a child’s caregiver(s) that they were making a report to child welfare, with participants stating they try to be “transparent” with families about their concerns. Participants found framing a referral to child welfare “as a support rather than an accusation,” (HCW-8) and stressing that they are mandated to report can help to maintain a positive therapeutic relationship (HCW-1).

#### 3.1.2. Theme 2: factors impacting healthcare providers’ confidence in reporting decisions

Healthcare providers generally reported feeling confident in their decision to make a report to child welfare when the concern clearly fell within their duty to report. One participant outlined this as “reasonable grounds to suspect that a child has been harmed or may be harmed based on the actions or inactions of a caregiver” (HCW-5). The participant went on to share that “once one has that concern for any reason…[their confidence in reporting] is there because the duty is so clear” (HCW-5). Specifically, participants reported that concerns involving hard evidence, such as injuries that lacked a “clear explanation” or were developmentally inappropriate, and instances of medical neglect clearly fell within their duty to report.

There were situations in which healthcare providers reported feeling more uncertain about their duty to report. As one participant stated, “there is that degree of uncertainty when it’s not… in your face assault, right? When it’s a bit more nuanced” (HCW-7). Participants indicated this complexity emerges specifically in complicated medical situations in which there are rare or unusual medical explanations that are difficult to confirm or cases with psychosocial complexities (e.g., Factitious Disorder Imposed on Another [FDIA], caregiver and adolescent conflict, and milder supervision concerns). Participants also reported that concerns about minimal child welfare response or lack of child or family benefit from child welfare involvement contributed to the feeling of uncertainty when reporting, particularly with these complex cases.

#### 3.1.3. Theme 3: areas for improvement

##### 3.1.3.1. Apprehension toward reporting

Understanding that child welfare involvement can be difficult and traumatic for families, healthcare providers reported sometimes feeling apprehensive about making reports. Healthcare providers highlighted how this knowledge often led them to weigh the costs and benefits of involving child welfare when deciding to make a report. One participant stated, “the challenge is deciding when I think…there’s enough risk to call, and you know deciding like what the trade-offs will be” (HCW-7). Another participant referred to child welfare as a “last resort [after] having exhausted…all the other reasonable and feasible steps” (HCW-3). Once a referral is made, participants feel child welfare workers do not appreciate the thought, time, or, at times, number of people involved in making the referral.

Further, healthcare providers reported fears of threats and legal retaliation from caregivers. Participants mentioned that there is always an “awareness” of potential retaliation from caregivers, and that it is an “ongoing reality” in their line of work. Participants also described specific incidents of receiving complaints or threats of lawsuits; some stated this fear is heightened when dealing with families who are “very confrontational” and “very resourceful.”

##### 3.1.3.2. Difficulty communicating with child welfare workers

Though healthcare providers shared that, generally, interactions with child welfare workers are positive, many explained that communication between the two professions can be difficult. In addition to scheduling issues, such as workers being busy or hard to reach, most participants reported that child welfare workers’ limited medical knowledge was a primary issue. One participant stated that, “the child protection team may not always have the knowledge around the medical situation in that it often takes a lot of convincing and education to relay the actual or potential concern” (HCW-2). Further, participants specifically referenced how they felt this difference in medical knowledge created different perceptions of risk between the two professions and made communicating their level of concern particularly challenging. One participant reported: “there have been times where it feels like we are kind of living in parallel universes… their perception of risk is so different…where it just feels like we are struggling to kind of connect in that way” (HCW-8).

##### 3.1.3.3. Disappointing response or outcome following referral

Many healthcare providers described occasions on which they found the child welfare response to their referrals unexpected, frustrating or disappointing, with results that were “not as protective as one would hope” (HCW-5). In particular, there were concerns that children and families were left without supports or services following case closure. One participant mentioned that “the level of risk has to get quite high before the response that you are hoping for is actually in place” (HCW-2). Another healthcare worker described a negative response from child welfare upon making a referral, where they felt the worker tried to discourage them from making the report because it was “messy” and complex (HCW-7).

##### 3.1.3.4. Lack of collaboration

Participants described a lack of collaboration between the healthcare and child welfare systems. While they understood that this was often due to confidentiality concerns, participants felt there were missed opportunities for healthcare providers to assist with cases. As one participant stated, “when there’s a really complex case where the medical team is so willing and able to really help the workers understand the issues, the worker instead just calls the family or shows up at the door, eliminating any opportunity for collaboration” (HCW-5). Many of the participants felt that improving collaboration between the two teams would benefit patients and their families.

##### 3.1.3.5. Disruption of therapeutic alliance

When asked about the impact calling CAS has on their relationship with families, some participants shared how the report can harm or negatively impact the relationship. One participant commented that, “once you call CAS the therapeutic alliance is kind of shot” (HCW-7). Participants cited the “lack of trust” following a report as being particularly detrimental. Participants noted that reporting tended to have a particularly negative effect when the caregivers felt they were being blamed or accused of harming their child. According to one participant, caregivers can take the report “very personally,” as though it was “an attack on them and their character” (HCW-1).

Multiple participants referenced relationships really suffering in the context of FDIA. FDIA (also referred to as Caregiver Fabricated Illness, medical child abuse, or Munchausen by Proxy, among other names) is a condition in which a caregiver induces or exaggerates an illness in their child so they receive ongoing medical care and treatment (26–28). As one participant stated, when a case involves FDIA “you know there’s already a bit of tension in the relationship… and then the call can…lead the relationship to deteriorate” (HCW-8). Participants noted that, since caregivers appeared well-intentioned in these cases, they were particularly difficult both to report to child welfare as well as for child welfare to intervene. Less commonly cited reasons for the breakdown of therapeutic relationships included having a family with a history of child welfare involvement and cases involving complex concerns.

#### 3.1.4. Theme 4: training

When asked about the level of child maltreatment training they received, all participating healthcare providers reported receiving very little formal training, unless they had specialized in child abuse pediatrics. One participant shared, “during training in medical school in general pediatrics, [child maltreatment is] a very small part of the core curriculum, but everyone has some degree of exposure. It’s minimal, it’s not at the level of an expert. It’s mainly focused on awareness and recognition” (HCW-5). Similarly for nurse practitioners, child maltreatment was a small component of the general training they received.

#### 3.1.5. Theme 5: professional roles

Many participants referenced the distinct roles of healthcare and child welfare professionals. One participant stated, “I think that we all have different roles… it’s understanding limits and boundaries and where your role as a physician starts and finishes” (HCW-3). Another worker reported, “I appreciate and understand that we are distinct entities with distinct expertise, and I have my role and they have their role” (HCW-5). Further, healthcare professionals understand that a key element of child welfare’s role is their ability to conduct investigations and gather information to determine “what is right for the child in those circumstances” (HCW 3).

#### 3.1.6. Theme 6: bias and systemic issues with access to support

The presence of systemic issues within both the healthcare and child welfare systems was often discussed. One participant mentioned the difficulty they had connecting families to community supports, while many others commented on the current lack of mental health supports available for children and caregivers. Participants acknowledged that many children and families with “multi-system” issues often “land on CAS’ lap” (HCW-4). Participants further noted that child welfare is often limited with regards to how much support they can offer to families or “what their response can be” (HCW-5). One participant indicated they felt the child welfare system is not designed to address structural barriers that exist for many of the children and families it serves.

Multiple participants raised concerns regarding biases and inequities present within child welfare based on the system’s history and current practices rooted in systemic racism. One participant stated, “in the past…there’s been some structural and systemic problems with how particular… populations or groups are treated [in child welfare]” (HCW-8). Another participant shared how a child’s background can impact a child welfare response, stating: “it is incredibly disheartening when you can anticipate what responses will be based on differing socioeconomic status and racial background” (HCW-5).

In particular, participants were concerned with the recent shift occurring in local child welfare practice toward a less invasive approach taken in investigations involving families of color in an effort to redress systemic overrepresentation of these children in child welfare systems. Healthcare providers reported concerns that children were harmed by the hesitant response of child welfare. One participant shared, “just as it is extraordinarily flat out wrong that some of the heavy-handed action from societies harmed children in the past, I also recognize that on this side there are children who are going to be harmed by inaction and a failure of society and institutions to protect them” (HCW-5). While participants stated they understood the reasons behind these policy changes (i.e., to reduce the number of children of color in care), they were worried for their patients.

Overall, participants highlighted the importance of the child welfare system being foremost oriented around protecting children and promoting their well-being. According to one participant, “the ideal is that the child welfare system is…an organization that’s just devoted to the welfare of children and that…accusation element [is] sidelined into one [small branch]… as opposed to taking over the entire like way people view the child welfare system” (HCW-8).

#### 3.1.7. Theme 7: suggestions for collaboration

When asked for suggestions on how to improve collaboration between the healthcare and child welfare systems, the most common answer was to improve communication between the two professions. While healthcare providers acknowledged the importance of protecting confidential information, participants noted that an “ongoing dialogue” would benefit children and families through increased knowledge-sharing and support. One healthcare worker mentioned that including child welfare workers in case conferences might help improve communication.

To address the concern of child welfare workers not understanding the medical science of certain cases, some participants suggested having specialized child welfare workers who deal with medical referrals. One participant believed having a group of workers in each agency that is familiar with child abuse pediatrics could help agencies understand healthcare professionals’ processes and medical decision-making factors. Similarly, another participant suggested there be more consistency with child welfare workers in cases to “have the least amount of transitions possible between worker and worker and worker” (HCW 2).

### 3.2. Child welfare workers

#### 3.2.1. Theme 1: healthcare professionals’ perceived expertise

When asked why child welfare investigations referred by healthcare professionals are more likely to involve more intrusive child welfare involvement and substantiation, many participating child welfare workers referenced healthcare providers’ perceived expertise and credibility. Some participants reported that healthcare professionals’ education and medical training better position them to recognize protection concerns. One participant emphasized this by stating that healthcare professionals “know what they are doing” (CW-13), while another stated: “we trust healthcare professionals in their jobs” (CW-12). Workers also identified the nature of protection concerns as an indicator of validation; healthcare settings reportedly see more severe cases of child abuse or neglect. As a result, the instances being reported to child welfare are more serious in nature and, therefore, more likely to be substantiated, opened for ongoing services, or involve a child welfare placement.

Some workers specified that the weight attributed to healthcare professional referrals has more to do with perceived expertise than actual credibility or family circumstances. One worker shared: “child welfare has historically… viewed the opinion of, you know, quote/un-quote professionals as more legit in comparison to community referrals or other referrals” (CW-11). According to some participants, regardless of the child protection concern, healthcare professionals are seen as reliable and credible sources that have more value attributed to their report due to their professional title.

#### 3.2.2. Theme 2: understanding the role of child welfare

Child welfare workers highlighted both positive and negative interactions with healthcare professionals, with many identifying healthcare providers’ understanding of child welfare’s role as the differentiating factor. Most participants stated that when healthcare professionals are more knowledgeable of child welfare workers’ responsibilities, jurisdiction, and capacity, the collaboration process is more pleasant. Too often, according to study participants, child welfare is reportedly contacted by healthcare professionals for matters that could be mitigated by other services. For instance, if a doctor is concerned about a caregiver’s mental health and not concerned about the safety or well-being of the child, child welfare workers reported it is more appropriate to make a referral to a community mental health service than calling a child welfare agency to make a report.

Many participants were confident that if more healthcare professionals better understood both the role of the child welfare system as a whole, as well as individual workers’ roles, both fields could engage in more effective work. One participant explained: “healthcare providers are not always informed as to how we do our work and what the process might be” (CW-3). Workers suggested healthcare professionals receive more training to help facilitate this understanding. Child welfare workers were also cognizant of the parallel learning process that should take place to create improved understanding. One participant reported: “if there was better understanding on both sides of the process…then it would be a better response [to child maltreatment concerns] and probably like a more unified one” (CW-13).

#### 3.2.3. Theme 3: need for increased collaboration

Though workers shared having positive experiences when collaborating with healthcare professionals, many reported there is room for improvement. Whether due to busy schedules or disinterest in engaging with child welfare workers after an initial report is made, healthcare professionals can be difficult to contact. Often, over the course of their investigations, child welfare workers need to speak with the healthcare professional who made the report to get details about the family and ask follow-up questions that only the reporting party can answer. As such, participants shared feeling frustrated by the lack of communication and partnership that can characterize interactions with healthcare professionals. According to participants, creating more effective collaborative relationships between the child welfare and healthcare systems starts with open communication. One worker shared: “the more communication that we have together, the more we work together as a team to support a family” (CW-4).

#### 3.2.4. Theme 4: systemic barriers and legacies of harm

Throughout the interviews, some workers expressed concern about child welfare’s legacy of harm in Canada and the impact it has on families. Participants reported that child welfare agencies are working hard to address the overrepresentation of Black and Indigenous children in care. The racism, biases, and assumptions that plague both the child welfare and healthcare systems are being challenged by child welfare workers. One participant stated: “[child welfare has] a well-known reputation. It’s not a good reputation. We’ve worked hard to earn it, but that does not mean that we have to fit into it” (CW-2). Child welfare workers believe that the process of challenging injustices and striving to create a more equitable system also needs to be initiated in healthcare settings to maximize the impact of these systemic changes. A worker reported: “it’s a whole new narrative that our agency is trying to bring about. So, I do not know what’s happening in the medical field, but that’s going to create quite a lot of barriers or issues if the healthcare system also does not choose to move forward with a new narrative” (CW-11).

Child welfare workers’ desire to see changes in healthcare professionals’ practices results from their concern for families that have had negative experiences with the healthcare system based on systemic inequities. One participant reported: “there is a certain middle class measuring stick that [healthcare professionals are] measuring their patients and our families up against. And if they do not meet that, they are very judgmental. They are very biased of different family situations” (CW-12). The expressed concerns extend beyond individual or personal biases, but rather shed light on the shortcomings of the healthcare system’s current structure. A child welfare worker shared: “I really worry about some healthcare settings being able to engage with certain families” (CW-2). To address the systemic barriers that limit families’ access to equitable and adequate service provision, participants strongly believed that changes must be made in both the child welfare and healthcare systems.

## 4. Discussion

By taking a multidisciplinary approach to data collection (i.e., by interviewing both healthcare providers and child welfare workers), we holistically examined the child welfare referral and investigation processes in situations where potential child maltreatment is identified in the healthcare setting. In doing so, we were able to identify both the strengths as well as the areas for improvement in the collaborative relationship between the healthcare and child welfare systems. Healthcare providers represent important points of contact for children as they routinely see vulnerable children including those too young to attend school and those with mental health concerns or disabilities (5, 29–32). Especially within the Canadian context of a universal healthcare system, children may be more likely to come into contact with healthcare professionals compared to other social systems and supports.

Our core thematic finding is that both groups felt the need for greater communication, collaboration, and understanding between the two professions. Although acknowledging that the healthcare and child welfare professions are demanding of workers’ time, both groups shared frustrations with the perceived lack of availability of their counterparts. It could be that explicit conversations around expectations with respect to level of involvement in the investigation process from the outset could help to mitigate some of the tension experienced between professionals during child welfare investigations. The two groups of participants described the importance of understanding each other’s professional roles. Appreciating the limitations of both their own and each other’s ability to assess and intervene with children and families was highlighted by study participants as a key facilitator of effective partnership. Overall, the participants indicated the importance of understanding not only the distinct roles of the two professions, but also acknowledging the limitations of their own expertise and appreciating what the other professionals can do that they cannot. Both child welfare workers and healthcare providers in this study highlighted the utility of having specialized teams that were familiar with the other profession to deal specifically with concerns of child maltreatment identified in a medical setting.

Findings from our study indicate that child welfare workers perceive healthcare providers to be expert assessors of potential child maltreatment. Paradoxically, healthcare providers in our study reported a lack of specialized training in child maltreatment accompanied by a frequent lack of confidence in identifying and reporting child maltreatment in cases that are not clear-cut cases of assault. This finding is consistent with findings from another study conducted with Canadian medical residents (16), and this dearth of training represents a significant gap within medical education and training.

Nonetheless, healthcare providers need to be attuned to signs of potential child maltreatment given their unique vantage point. Early identification and thoughtful intervention have the potential to mitigate some of the downstream effects of child maltreatment. The logical first step in addressing suspected child maltreatment is making a referral to child welfare services, where trained professionals can further investigate and intervene where necessary. Indeed, participating healthcare providers cited benefits of reporting child maltreatment. Yet, consistent with findings of previous studies, healthcare providers in our study highlighted several barriers to reporting suspected child maltreatment including fear of disrupting the therapeutic relationship with the child/family, causing increased harm to the family through involving child welfare, and being threatened with legal retaliation from the family (12–14).

While healthcare providers in our study indicated there are certain circumstances in which they are more certain that a referral to child welfare should be made, they report hesitancy in other situations. As was found in other studies, healthcare providers indicated that the decision to refer to child welfare is clear in cases with evidence of physical or sexual abuse that squarely fall within the mandate to report (12, 21). On the other hand, the decision to report is more nuanced in cases with more complex medical explanations of injuries/presenting concerns or those with other elements at play such as FDIA, caregiver/teen conflict, and some supervision concerns. Extant literature similarly states that healthcare professionals tend to hesitate to refer to child welfare in cases with increased complexity (16, 21). Oftentimes, healthcare providers attempt to mobilize supports within the healthcare setting and consult many different members of the healthcare team before making a report, as they recognize and appreciate the repercussions their report may have for the family. This could be especially true in these more complex cases where the duty to report is less clear-cut.

Systemic barriers to accessing equitable care and services along with legacies of harm within both the child welfare and healthcare systems were brought up by interview participants. In particular, they spoke of the overrepresentation of Indigenous and Black children in the child welfare system. While participating child welfare workers referred to efforts being made to redress this overrepresentation, healthcare providers identified limits to these efforts and identified possible harms with inaction. Participants identified further biases against children and families with lower socioeconomic status. Ontario child welfare-involved children experiencing economic hardship are more likely to be struggling in multiple domains including having developmental concerns and academic difficulties (33). Further, these children are more likely to be involved in substantiated child welfare investigations (33). The fact that participants identified problematic responses to families struggling with economic hardship likely represents strongly embedded societal biases against these families along with systemic factors that have left them more vulnerable to situations that might warrant child welfare involvement. Other systemic concerns including access to services were brought up by participants; these represent ongoing issues with supports available to families (34–36).

### 4.1. Limitations

Some limitations should be considered when interpreting study results. The sample consisted of 13 child welfare and eight healthcare providers from two Ontario child welfare agencies and one large pediatric tertiary care center. The use of snowball sampling methodology resulted in the selection of participants with similar areas of expertise. That said, we did not collect information on healthcare providers’ sub-specialties beyond pediatrics. It was revealed through the interviews, though, that a large proportion of the healthcare providers who participated in our study worked specifically in child abuse pediatrics meaning they had unique perspectives even when compared to other pediatric healthcare providers. In general, the use of a voluntary sample could have selected for individuals with more knowledge on or interest in the study subject matter or those with particularly positive or negative experiences with the child welfare system. One participant declining to consent to audio recording and the use of the transcription function, and an audio technical error in a separate interview decreased confidence in the quality of the transcription of those two interviews.

## 5. Conclusion

To our knowledge, this is the first study that incorporates voices from both the child welfare and healthcare systems in an attempt to identify areas for improvement and strengths in collaboration between these professional groups. Healthcare providers remain an important point of contact for vulnerable children and their families to access necessary services that can support and protect them, including child welfare services. That said, child welfare involvement can be very disruptive to the family unit, and our study findings demonstrate that healthcare providers do not make the decision to report to child welfare lightly. Ideally, the child welfare and healthcare systems would be complementary to each other and work synchronously to best support children and families. Unfortunately, the results of our study identify barriers to this collaborative work, including inadequate communication and understanding between the professions, hesitations in healthcare professionals’ reporting, along with legacies of systemic injustices in both sectors. Several future steps were identified that might help promote improved collaboration and, therefore, more streamlined and effective services provided to children and families. Future research might include focus groups combining professionals from both groups to encourage specific interactions and feedback about how to ethically improve collaboration. Future research might also expand to include family doctors who have a different perspective compared to pediatricians. Ultimately, best practices for education, training, and collaboration in both professions need to be ascertained to promote optimal outcomes for children and families.

## Data availability statement

The original contributions presented in the study are included in the article/supplementary material, further inquiries can be directed to the corresponding author.

## Ethics statement

The studies involving human participants were reviewed and approved by University of Toronto Ethics Board. The patients/participants provided their verbal informed consent to participate in this study.

## Author contributions

NJ-C and BF conceptualized the study. EL, MK-C, and NJ-C conducted the interviews, performed the interview coding, and drafted the initial manuscript. AV, JS, and DL assisted with developing initial interview questions. NJ-C, BF, EL, MK-C, AV, and JS drafted the ethics documents, recruited the participants, and reviewed all interview codes. BF obtained the funding. All authors approved the final manuscript.

## Funding

The manuscript was completed with financial support from BF’s Social Sciences and Humanities Research Council of Canada Research Chair in Child Welfare (#950–231186).

## Conflict of interest

The authors declare that the research was conducted in the absence of any commercial or financial relationships that could be construed as a potential conflict of interest.

## Publisher’s note

All claims expressed in this article are solely those of the authors and do not necessarily represent those of their affiliated organizations, or those of the publisher, the editors and the reviewers. Any product that may be evaluated in this article, or claim that may be made by its manufacturer, is not guaranteed or endorsed by the publisher.

## Appendix

### Appendix. Interview questions

#### Healthcare providers

1. How long have you been working as a healthcare provider?
2. What kind of training, if any, have you received for assessing child maltreatment?
3. Over the course of your career, have you noticed signs of child maltreatment or been concerned that a child is at risk for maltreatment? *If no, do not conduct interview.*
  a. If yes, describe the most recent referral you made to child welfare.
4. Describe a time where you were very certain in your decision to make a report to child welfare.
5. Describe a time when you were uncertain in your decision to make a report to child welfare.
6. Have you ever regretted your decision to make a report to child welfare? If so, please describe.
7. Describe a time when your report to child welfare positively impacted your relationship with a family.
8. Describe a time when your report to child welfare negatively impacted your relationship with a family.
9. Describe what, if any, supports you would offer to a family in the following scenarios:
  a. You suspect the family is experiencing economic hardship.
  b. You suspect concerns for the caregiver (e.g., caregiver has substance abuse concerns, has mental health concerns, is a victim/perpetrator of intimate partner violence, or lacks social supports).
  c. You suspect concerns for the child’s functioning (e.g., developmentally, behaviourally, academically, etc.).
  d. You have a strong suspicion of abuse or neglect.
10. In general, how would you describe your experiences with the child welfare system?
11. How do you expect the child welfare system to support a family you refer?
12. Do you have any suggestions for how the healthcare and child welfare systems could work together to better support children and families?

#### Child welfare workers

1. Are you a screening or investigating worker?
2. How long have you worked as a (screening worker or investigating worker)?
3. In the past year, have you received a referral or investigated a case that was referred from a healthcare worker?
4. Data from the Ontario Incidence Study of Reported Child Abuse and Neglect 2018 indicate that investigations involving healthcare referrals are more likely to be substantiated, opened for ongoing services, or involve a child welfare placement compared to other referral sources. Do you have any insight as to why this would be the case?
5. How do you think healthcare providers can best support a family prior to making a referral to child welfare?
6. Overall, how would you describe your interactions with healthcare professionals in the context of a child welfare referral or investigation?
7. Do you have any suggestions for how the healthcare and child welfare systems could work together to better support children and families?
